# Genome Mining and Comparative Genome Analysis Revealed Niche-Specific Genome Expansion in Antibacterial *Bacillus pumilus* Strain SF-4

**DOI:** 10.3390/genes12071060

**Published:** 2021-07-12

**Authors:** Sajid Iqbal, John Vollmers, Hussnain Ahmed Janjua

**Affiliations:** 1 Department of Industrial Biotechnology, Atta-Ur-Rahman School of Applied Biosciences (ASAB), National University of Sciences and Technology (NUST), H-12 Islamabad 44000, Pakistan; siqbal.phdabs12asab@asab.nust.edu.pk; 2 Institute for Biological Interfaces 5 (IBG-5), Karlsruhe Institute of Technology (KIT), 76344 Eggenstein-Leopoldshafen, Germany; Jhon.vollmers@kit.edu

**Keywords:** *Bacillus pumilus*, whole-genome sequence, biosynthetic gene clusters (BGCs), plant growth-promoting bacteria, phylogenetic and comparative genome analysis, genome plasticity

## Abstract

The present study reports the isolation of antibacterial exhibiting *Bacillus pumilus* (*B. pumilus*) SF-4 from soil field. The genome of this strain SF-4 was sequenced and analyzed to acquire in-depth genomic level insight related to functional diversity, evolutionary history, and biosynthetic potential. The genome of the strain SF-4 harbor 12 Biosynthetic Gene Clusters (BGCs) including four Non-ribosomal peptide synthetases (NRPSs), two terpenes, and one each of Type III polyketide synthases (PKSs), hybrid (NRPS/PKS), lipopeptide, β-lactone, and bacteriocin clusters. Plant growth-promoting genes associated with de-nitrification, iron acquisition, phosphate solubilization, and nitrogen metabolism were also observed in the genome. Furthermore, all the available complete genomes of *B. pumilus* strains were used to highlight species boundaries and diverse niche adaptation strategies. Phylogenetic analyses revealed local diversification and indicate that strain SF-4 is a sister group to SAFR-032 and 150a. Pan-genome analyses of 12 targeted strains showed regions of genome plasticity which regulate function of these strains and proposed direct strain adaptations to specific habitats. The unique genome pool carries genes mostly associated with “biosynthesis of secondary metabolites, transport, and catabolism” (Q), “replication, recombination and repair” (L), and “unknown function” (S) clusters of orthologous groups (COG) categories. Moreover, a total of 952 unique genes and 168 exclusively absent genes were prioritized across the 12 genomes. While newly sequenced *B. pumilus* SF-4 genome consists of 520 accessory, 59 unique, and seven exclusively absent genes. The current study demonstrates genomic differences among 12 *B. pumilus* strains and offers comprehensive knowledge of the respective genome architecture which may assist in the agronomic application of this strain in future.

## 1. Introduction

Soil salinity, iron/phosphorus deficiency, and drought stress are the major problems that can limit plant growth and its associated products [1]. Certain beneficial soil bacteria could play a significant role in iron/phosphorus deposition, drought, and salinity stress and hence can promote plant growth and crop yields [2,3]. Plant growth-promoting bacteria establish specific symbiotic interactions with plants and colonize intracellularly or intercellularly without causing any infection. These bacterial strains could be used in biocontrol, bio-fertilization, and biostimulation to improve plant growth under various harsh conditions [4]. *Bacillus* spp. are important rhizosphere bacteria that may facilitate plant growth and crop yields through various mechanisms [5]. For instance, in iron-deficient soil, plants like red clover recruit certain types of rhizosphere bacteria with greater capacity for siderophore synthesis. Bacterial siderophore production is commonly associated with increased iron acquisition in plants in calcareous soil where most of the iron is in an unavailable state [6]. Over the past few years, bio-control of plant pathogens are significantly increased due to the adverse effect of chemical control on soil fertility and ecology [7]. Therefore, the focus of recent research is switched towards alternative strategies that employ potential bacteria for biocontrol.

*B. pumilus* is a Gram-positive, aerobic, spore-forming bacteria that produces multifarious metabolites and exhibits increased resistance to biotic and abiotic stress. They are found in diverse environments from soil to living organisms and from air to deep-sea sediments [8]. *B. pumilus* isolated from plant root regions exhibited potential plant growth-promoting properties [9]. Whereas *B. pumilus* strains isolated from shrimp exhibited antibacterial activity against marine pathogens [10]. Bacterial secondary metabolites are not only important for the producer cells but also have an impact on their host. These metabolites have significant applications in agriculture and pharmaceutics as bioactive compounds [11]. Advances in sequencing technologies and the development of robust genome mining tools enable researchers to uncover the molecular basis of the strain versatile lifestyle and prioritize industrially important secondary metabolites at the genome level. These specialized metabolites represent an untapped resource for viable crop yield and antibacterial agents. For instance, previously genome mining of *B. pumilus* strain TUAT1 reported that it encodes several plant growth-promoting genes involved in indole 3 acetic acid synthesis and acetone metabolism [12].

The current study was aimed to explore the cryptic biosynthetic potential of antibacterial exhibiting *B. pumilus* isolate that could be used as a biocontrol agent. In this regard, we isolate and sequenced the genome of antibacterial exhibiting *B. pumilus* strain SF-4 and performed comprehensive genome analysis along with all publicly available whole-genomes of *B. pumilus* (June 2020). The study revealed distinguish biosynthetic potential among *B. pumilus* strains and niche-specific adaptation was detected. Moreover, the in-vitro antibacterial activity and plant growth-promoting genes in the strain SF-4 genome provide a firm foundation for its application as a biocontrol agent.

## 2. Materials and Methods

### 2.1. Soil Sampling, Isolation, and Antibacterial Activity

*B. pumilus* strain SF-4 was isolated from a soil sample collected from an arid soil field District Karak (33°1105 N, 71.0914 E), Khyber Pakhtunkhwa Pakistan. District Karak is located in the southern region of Pakistan and lies between 32°48′ to 33°23′ North latitudes and 70°40′ to 71°30′ East longitude. This region is highly rich in terms of natural resources such as uranium, salt, and natural gas. The majority of the area is arid and the average annual precipitation is 330 mm [13]. The sample was serially diluted and 200 µL from each dilution suspension was inoculated on tryptic soya agar (TSA) plates having cycloheximide (100 µg/mL) as an antifungal agent and incubated at 37 °C for 48 h. Single colonies were obtained using repeated subculturing techniques. The antibacterial activity of strain SF-4 was evaluated against a set of Gram-positive and Gram-negative American Type Culture Collection (ATCC) bacterial strains (*Streptococcus pneumoniae* 6305, *Shigella flexneri* 12022, *Klebsiella pneumoniae* 13889, *Staphylococcus aureus* 6538, *Escherichia coli* 8739, *Salmonella typhimurium* 14028, *Listeria monocytogenes* 13932, and *Pseudomonas aeruginosa* 9027) on Muller-Hinton agar (MHA) plates. The strain SF-4 was inoculated on MHA in the middle of an agar plate followed by incubation at 37 °C for 48 h. Subsequently, the plate was subjected to chloroform for 8 h to kill bacteria and the plates were placed in a fume hood to evaporate the solvent. The second layer of semi-solid nutrient agar (0.7% agar) culture containing 10^2^ colony-forming unite/mL of indicator strain was poured and incubated overnight at 30 °C, the antibacterial activity was observed as a halo zone on the plate [14].

### 2.2. Genomic DNA Extraction and Strain Identification

The strain SF-4 was grown in tryptic soy broth (TSB) at 37 °C for 18 h. Total genomic DNA was extracted using the Purlink Genomic DNA extraction kit (Invitrogen, Carlsbad, CA, USA), according to the manufacturer’s instructions. The integrity and quantity of gDNA was confirmed through gel electrophoresis (0.8% Agarose) and NanoDrop (Titertek Berthold, Germany), respectively.

### 2.3. Whole-Genome Sequence, Assembly, and Annotation

Genomic DNA library was prepared using a Nextera XT library preparation kit (Illumina Inc. SDCA, USA) and sequenced using Illumina Hiseq 2500 platform with an end paired approach and 250 cycles per reads. Whole genome sequencing was performed by MicrobesNG (University of Birmingham, Birmingham, UK). Reads were trimmed using trimmomatic v 0.36 [15] and de novo assembly was performed using SPAdes v 3.12 [16]. Gaps within the scaffolds were filled as described earlier [17] and genome annotation was performed using NCBI Prokaryotic Genome Annotation Pipeline (PGAP) v.4.10 [18]. The draft genome sequence of *B. pumilus* strain SF-4 was submitted to NCBI, Genbank under the accession number CP047089.1. The BioSample and BioProject were registered under accession numbers SAMN13526181 and PRJNA594265, respectively. The final annotated genome was visualized using CG viewer to show genes location, GC skew, and GC content (http://stothard.afns.ualberta.ca/cgview_server/, accessed on 27 January 2021).

### 2.4. Genome Mining

The putative BGCs were identified using web-based Antibiotic and Secondary Metabolite Analysis SHell (AntiSMASH) 5.0 (https://antismash.secondarymetabolites.org/, accessed on 6 January 2021). Additionally, both ‘known clusters’ and ‘unknown cluster’ blast modules were selected to find similar clusters by genome comparison. Sequence similarities to known clusters and domain functions were predicted and annotated using BLASTp and pfam analysis [19]. Gene functions and subsystem categories were predicted using Rapid Annotation using Subsystem Technology (RAST) and SEED server [20].

### 2.5. Identification of Putative Horizontal Gene Transfer (HGT)

Genomic islands (GIs) are regions within the genome that typically are 10–200 kb in length and have been acquired through HGT [21]. GIs play an important role in adaptation, evolution, and carrying genes that are associated with metabolism, antibiotic resistance and symbiosis. To predict GIs, the annotated *B. pumilus* SF-4 genome was submitted to Islandviewer 4 online server (https://www.pathogenomics.sfu.ca/islandviewer/, accessed on 6 January 2021) using *B. pumilus* SAFR-032 as a reference genome. Islandviewer 4 predicts GIs in bacterial and archaeal genome using three prediction methods: IslandPath-DIMOB, IslandPick, and SIGI-HMM. Moreover, PHASTER server (https://phaster.ca/, accessed on 5 January 2021) was used to identify prophage regions in the SF-4 genome.

### 2.6. Comparative Genome Analysis

To determine diversity and strain-specific features in *B. pumilus* genomes, the bioinformatics pipeline “Bacterial Pan-Genome Analysis” (BPGA) was employed [22]. Although 83 genome assemblies of *B. pumilus* strains are currently available in public databases, most of these are incomplete draft genomes. Therefore, only complete genomes or chromosomes were selected and utilized for comparative genome analysis. The annotated 11 complete genomes downloaded from the NCBI database and one newly sequenced genome were used as input for the BPGA pipeline. The BPGA orthologous cluster analysis results based on the 12 *B. pumilus* genomes were used as input for the clustering of genes into families through USEARCH with a sequence similarity cut-off value of 0.5. The size of the core genome was determined as the number of common gene families shared by all analyzed genomes while the pan-genome size was defined as the sum of all gene families [23]. The core, accessory, and unique gene sets were extracted using the pan-genome extraction module of BPGA. Phylogenetic analyses were conducted based on concatenated core genes alignment using a binary pan gene matrix (presence/absence of each gene family across the genomes) and a maximum likelihood (ML) tree was constructed using MEGAX [24]. Furthermore, CSIphylogeny 1.4 (https://cge.cbs.dtu.dk/services/CSIPhylogeny/, accessed on 25 July 2020) was utilized to construct whole-genome single nucleotide polymorphism (wgSNP) based phylogenetic tree. CSI phylogeny, determine and validates SNPs using BWA and filters the SNPs observed within 10 base pairs. The SNPs are aligned and construct ML tree. The position where no SNPs are found or where SNPs have been ignored are considered identical to the base in the reference genome.

To assess the diversity in *B. pumilus* strains, high throughput average nucleotide identity (ANI) analysis was performed [25]. The resulting ANI distance matrix was visualized using an online heatmapper tool (http://www.heatmapper.ca, accessed on 28 March 2021). In-silico DNA–DNA hybridization (DDH) was made for species delineation using the genome-to-genome distance calculator GGDC-2.1 online web server (https://ggdc.dsmz.de/ggdc.php#, accessed on 9 January 2021). The GGDC is a state-of-the-art in-silico technique for genome-to-genome comparison and more precise as compared to the conventional DDH technique. Genome-wide annotation and comparison of clusters of orthologous groups (COGs) were performed using the web-based tool orthoVenn2 [26]. The completeness and quality of the genomes were estimated using CheckM tool [27].

## 3. Results and Discussion

### 3.1. B. pumilus SF-4 Isolation and Antibacterial Activities

The here sampled arid habitats offer a special ecosystem due to relatively high temperature, low water content, and high radiation. Therefore, diverse bacterial strains with uncommon metabolic activities are expected [28]. *Bacillus* spp. are a predominant group of soil bacteria due to their abilities to form endospores and antimicrobial metabolites [29]. In the present study, a total of 109 *Bacillus* spp. were isolated from various arid soil areas and evaluated for antagonistic activities. Of these, 16 isolates showed mild to strong antibacterial activities against at least two indicator strains in preliminary screenings. Among these strains, SF-4 exhibited promising antibacterial activities against all indicator strains and was selected for further analysis. The strain SF-4 exhibited higher activity against *P. aeruginosa* and *S. flexneri* while it showed the lowest activity against *S. typhimurium* (Supplementary File 1, Figure S1). This is in agreement with a previous study where *B. subtilis* RLID 12.1, isolated from soil, inhibited the growth of both Gram-positive and Gram-negative bacterial strains [30]. Previous studies report that several plant diseases can be controlled by natural antagonistic microbes [31,32]. Such antagonistic microbes can establish a composite relationship with plant pathogens and may be involved in the production of antimicrobial metabolites, the competition for space and nutrients, or the activation of plant defense mechanisms [33].

### 3.2. Genomic Features of B. pumilus SF-4

**Figure 1:**
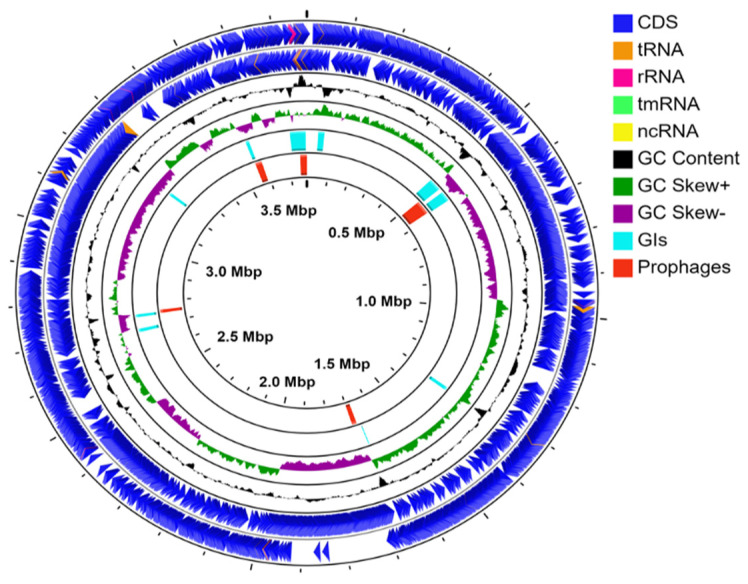
Circular visualization of *B. pumilus* strain SF-4 genome. Circle from outside to inside represent the position of protein-coding genes (CDS), tRNA, rRNA genes on the positive (circle 1) and negative strands (circle 2). Circle 3 (green and purple) and 4 (Black) show GC skew and GC content plotted as the deviation from the average for the complete genome. Circle 5 and 6 represent the genomic position of putative GIs (light blue) and prophages (red), respectively.

The *B. pumilus* SF-4 genome is 3,774,709 bp in size with 41.18% GC content. A total of 3844 genes, 3754 coding DNA sequences (CDSs), 75 tRNAs, 10 rRNAs, and 5 ncRNAs were identified. The relative genomic positions of protein-coding sequences, rRNA, tRNA genes, and GC skew are shown in Figure 1. The draft genome of SF-4 contains 93 contigs with 44× coverage and N50 and L50 were calculated as 296,073 and 5, respectively. Gaps within the scaffold were closed and contigs less than 500 bp were excluded which result in a high-quality, single scaffold non-circular genome with 96.36% completeness. Core genome phylogeny, ANI score, and whole-genome SNP analysis collectively indicate that strain SF-4 is closely related with *B. pumilus* SAFR-032 and *B. pumilus* 150a which were previously isolated from spacecraft and top sediment layers, respectively.

### 3.3. Genomic Islands (GIs) and Prophages

A total of 10 GIs were identified in *B. pumilus* SF-4 genome ranging from 4384 bp to 54,694 bp in size (Figure 1). Of the total 232 CDS located in these GIs, 70 encode “hypothetical proteins” with unknown function while the rest are mainly associated with carbohydrate metabolism and stress response. These genes are prominently related to bacillithiol biosynthesis, metallo-hydrolase, iron chaperone, peptidases M15, phage holing, glycosyltransferase, UV damage repair protein, collagen-like protein, α-D-glucose phosphate-specific phosphoglucomutase, and zinc-binding dehydrogenase (Supplementary File 2 Table S1). These findings indicate that numerous genes in strain SF-4 are likely to be acquired through HGT and are in agreement with an earlier genome analysis of *S. thermosulfidooxidans* and *Sinorhizobium* species [34,35]. Moreover, the presence of these genes in GIs indicates that HGT provides an additional advantage for metabolic diversity and coping with stress conditions. Phage screenings with PHASTER identified four prophage insertions, among which one is intact and three were identified as incomplete prophages in the *B. pumilus* SF-4 genome (Figure 1). The intact prophage is 59.9 kb in size and consists of 73 CDS. Of these, 17 represent ‘hypothetical proteins’ with unknown function and 50 are common phage-related proteins such as capsid, head, tail, terminase, integrase, etc. A detailed sequence analysis of the intact phage revealed significant similarity with Brevibacillus phage Jimmer 1 (NC_029104) while incomplete prophage regions 2, 3, and 4 exhibited similarity with Bacillus phage SP-10 (NC_019487), Brevibacillus phage Jimmer 1 (NC_029104) and staphylococcus SP-β like phage (NC_029119), respectively (Table 1).

### 3.4. Pan-Core Genome Analysis

**Figure 2:**
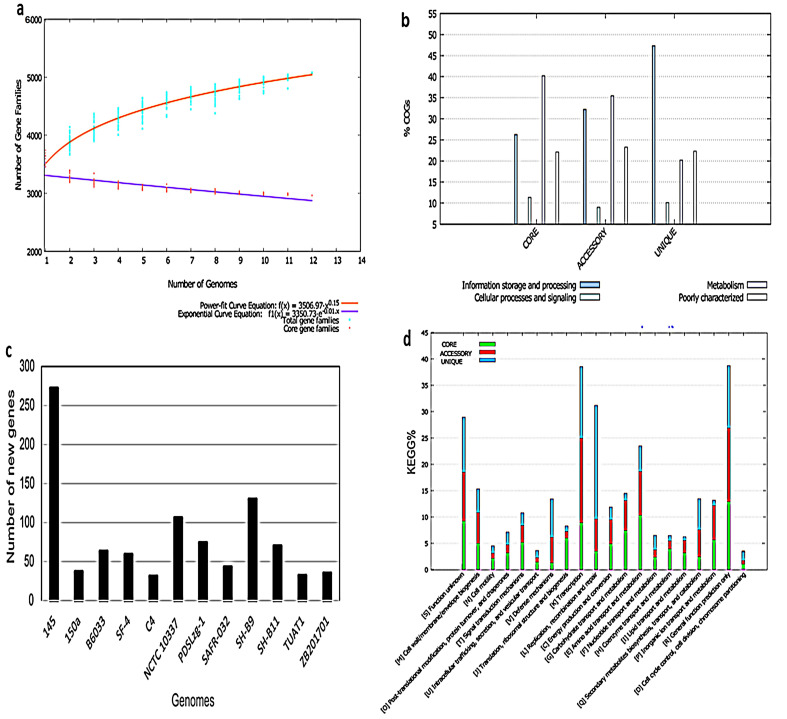
Pan-genome analysis of all *B. pumilus* strains (*n* = 12). (**a**) Represent the core and pan-genome plot. (**b**) The cluster of orthologous groups (COGs) distribution of representative genes in the unique, core, and accessory genome (**c**) shows the number of new genes added to each genome. (**d**) Kyoto encyclopedia of genes and genomes (KEGG) distribution in the unique, accessory, and core genomes.

The currently available complete genome and chromosomes in public databases represent isolates obtained from various ecological niches (Table 2). Herein, we aimed to identify accessory and unique genes in *B. pumilus* strains that may contribute to their adaptation in specific environmental conditions. We defined a ‘pool genome’ by selecting available complete genomes (*n* = 10) and chromosomes (*n* = 2) and divided them into accessory, core, and unique genes. The core genome consisted of 2962 genes for all selected genomes, while the pan-genome was determined to be ‘open’ for expansion (Figure 2a). The pan-genome of *B. pumilus* additionally contained 452 to 603 accessory genes in all analyzed strains (Figure 2b). Furthermore, a total of 952 unique genes and 168 exclusively absent genes were prioritized across the genomes. While *B. pumilus* strain SF-4 genome consists of 520 accessory genes, 59 unique genes, and 7 exclusively absent genes. Intriguingly, secondary metabolites highlight ecological niche-specific adaptation in the strain. Genes involved in information storage and processing, cellular processing, signaling, and metabolism were identified among the unique, accessory, and core genomes are shown in Figure 2c,d. The details of accessory, core, and unique genes in *B. pumilus* strains are given in Supplementary File 2, Table S2.

**Figure 3:**
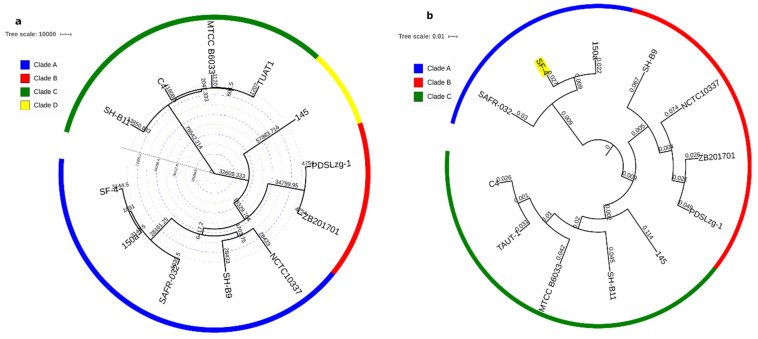
(**a**) Phylogenetic tree based on whole-genome SNP of *B. pumilus* strains, rooted with *Bacillus cereus* (accession number AE016877) as an outgroup which was removed from the final figure. The length of the scale bar indicates nucleotide substitution 10,000 sites. Branch length is proportional to the numbers of SNPs that are given above each branch (**b**). The core phylogeny tree was constructed based on 2962 concatenated core proteins of 12 *B. pumilus* genomes. The number alongside each branch represents the time of divergence (Million years ago). The trees were generated by the maximum likelihood (ML) method, in MEGAX and were edited in iTOL (https://itol.embl.de/, accessed on 28 February 2021).

**Figure 4:**
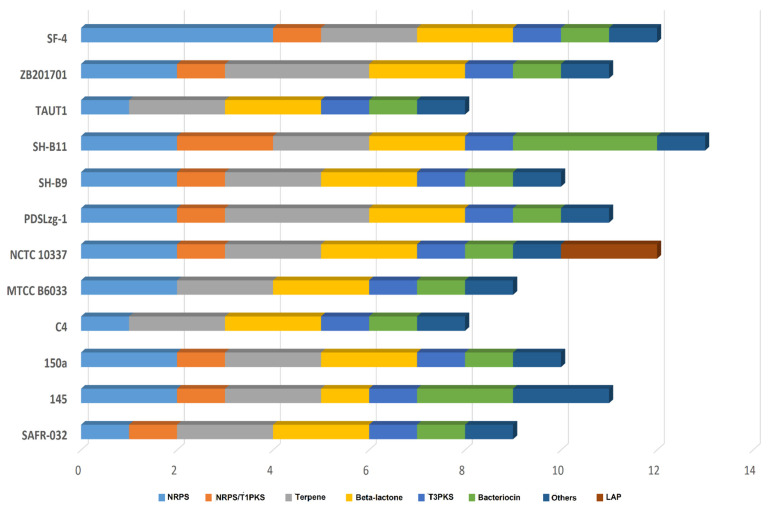
Distribution of biosynthetic gene clusters (BGCs) in 12 *B. pumilus* strains. BGCs are color-coded as per legend. The distribution depicts that strain SH-B11, NCTC10337, and SF-4 harbor the highest number of BGCs in their genomes.

Phylogenetic analyses based on whole-genome SNPs and concatenated 2962 core protein sequences indicate local diversification in the selected strains (Figure 3a,b). The 12 analyzed genomes could be clustered into at least three distinct clades. In the whole-genome SNP tree, clade A consists of five strains—i.e., SF-4, SAFR-032, 150a, SH-B9, and NCTC10337. Clade B and C comprised of four (C4, TUAT1, MTCC B6033, SH-B11) and two strains (ZB201701 and PDSLzg-01), respectively while strain 145 expanded separately (Figure 3a). The diversification of strain 145 is probably linked to its large genome size as compared to other *B. pumilus* strains. Already earlier, larger genome sizes within species was linked with gene acquisition via HGT which alters the evolutionary dynamics and expands the adaptive potential [36]. We also observed that strain 145 carries the highest number of new genes which further support HGT events and diversification (Figure 2c). Comparative genome analysis of plant-associated and non-plant-associated *Bacillus* spp. revealed that plant-associated strains harbor more genes relevant to secondary metabolites biosynthesis and also carry a higher number of unique genes as compared to non-plant-associated strains. Furthermore, HGT analysis confirmed that most of the genes were acquired by plant-associated strain during the evolutionary process [37]. Also, a phylogenetic tree based on the core genome illustrated 3 clades and strain 145 is grouped with C4, TUAT1, MTCC B6033, and SH-B11 in clade C (Figure 3b). The newly sequenced strain SF-4 clusters within clade A with reference strains SAFR-032 and 150a, which were isolated earlier from spacecraft and sediment top, respectively. It was noted that the biosynthetic potential and unique genome pool of these strains diverge (Figure 4) which suggests that some important changes in their genomes may have occurred during the adaptation to their respective habitat [38]. This implies that specific habitats have profound effects on HGT and highlights uniformity between nucleotide sequence and hierarchal clustering [39], in agreement with published data that demonstrates lateral gene transfer within various *streptococcus* species sharing the same habitat [40].

### 3.5. Unique Gene Pool in B. pumilus Strains

The here observed strain-specific genes fall into different functional categories. A high proportion of strain-specific genes are associated with “replication, recombination and repair” (L), transcription (K), and “general function prediction only” (R) (Figure 2e). The number of unique genes in various strains ranges from 31 to 272 with the fewest identified in strains C4 and most in strain 145 (Figure 2d). Gramicidin biosynthesis, autolysin, and restriction endonuclease encoding genes were identified among unique genes of strain 145 which was isolated from a sediment surface. Strain C4 contains type I restriction endonuclease subunit R coding genes. Exclusive gene functions for strain 150a included serine proteases while strain B6033 genes encoding for ABC transporter permeases, RecQ family DNA helicase, phosphotransferase system transporters, restriction endonucleases, as well as IS3 family transposases were observed to be unique for this strain. Serine proteases are known to enhance the survival of producer organisms in stress conditions while restriction endonucleases are involved in defensive mechanisms and cleave DNA that is foreign to the bacterial cells [41]. The reference strain NCTC 10,337 was found to include KR domain-containing protein peptidase G2, thiazole/oxazole modified microcins (TOMM) precursor leader peptide-binding proteins, putative thiazole-containing bacteriocin protein, and YcaO-like family proteins among its unique genes. A recent study demonstrated YcaO and TOMM cyclodehydration and peptide recognition during the biosynthesis of azoline which inhibits the growth of fungi [42]. The strain PDSLzg-1 carries unique genes that encode for glycosyltransferase, polyribitol phosphotransferase, AAA family ATPase and type III-B CRISPR module RAMP protein Cmr1. The phosphotransferase system is a process used by various bacterial species for sugar uptake where the source of energy is from phospho-enol-pyruvate [43].

The reference strain SAFR-032 includes DEAD/DEAH box helicases, glycosyltransferases, DNA cytosine methyltransferases, ABC transporter permeases, ATP binding proteins, “ATP-grasp domain-containing” proteins, patatin-like phospholipase family proteins and dTDP-glucose 4,6-dehydratase among the predicted functions of its unique genes pool. These proteins are primarily associated with metabolic pathways. For instance, ATP-grasp domain-containing protein is a superfamily of proteins that contain an atypical ATP binding site and involved in several metabolic pathways like gluconeogenesis and fatty acid synthesis [44], while DEATH box proteins are associated with an assortment of metabolic pathways that typically involve RNAs [45]. The strains SH-B9 and SH-B11 which were isolated from sugar beet rhizosphere, exclusively contain genes encoding for AAA family ATPase, DNA cytosine methyltransferase, nucleotidyltransferase, restriction endonuclease subunit S, ParM/StbA family protein, and transglycosylase SLT domain-containing protein, N-6 DNA methylase, ArsR family transcriptional regulator, group II intron reverse transcriptase, DNA recombinase, caspase family protein, dNTP triphosphohydrolase, radical SAM protein, and major facilitator superfamily (MFS) transporter. MSF is the largest known family of secondary active transporters and plays a key role in many physiological processes [46].

The strain TUAT1 contains trypsin-like serine protease and YukJ family protein while strain ZB201701 contains type I restriction-modification system, LLM class flavin-dependent oxidoreductase, 3-hydroxybutyrate dehydrogenase, oxygen-insensitive NADPH nitroreductase, hydrolase, restriction endonuclease subunit S, and response regulator transcription factor. Both hydrolase and 3-hydroxy isobutyrate dehydrogenase is associated with stress condition [47,48]. Several studies have reported that HGT occurs between various species of bacteria and plays an important role in the development of drug-resistant and physiological fitness in a specific niche [49]. It is therefore likely that a large number of unique genes in each strain were acquired through HGT from co-existent species in a particular niche.

The unique genes in strain SF-4 encode for various lipopeptide biosynthesis (amino acid adenylation domain-containing protein), recombinase family protein, collagen-like protein, TetR family transcriptional regulator, tyrosine-type recombinase/integrase, DNA cytosine methyltransferase (Supplementary File 2 Table S3). Collagen-like protein encoding genes were identified earlier in several bacterial strains and were found to be associated with producer strain survival in extreme environments [50]. Furthermore, these unique genes displayed significant divergence from the original strain GC content which indicates the acquisition via HGT. A large portion of unique genes in strain SF-4 genome encode hypothetical proteins with unknown function. Therefore, these genes may be associated with novel biosynthetic commensal interaction in a specific habitat. However, several genes identified that enable the strain to interact with its natural environment and contribute to its fitness.

### 3.6. Comparison of BGCs in B. pumilus Strains

*B. pumilus* strain SF-4 genome is rich in terms of BGCs and produces several beneficial secondary metabolites. A total of 12 BGCs—including four NRPS, two terpenes, and one each T3PKS, hybrid (NRPS/PKS), lipopeptides, β-lactone, bacteriocin, and other clusters (secondary metabolite like protein)—were identified in the *B. pumilus* strain SF-4 genome (Figure 4). 

Majority of the predicted BGCs presented low levels of similarity with known clusters while four BGCs encoding RiPP-like, β-lactone, T3PKS, and terpene did not show any similarity with known clusters (Supplementary File 2 Table S4), suggesting that these clusters may code for new natural products. Most notably, at least two terpenes and β-lactone and one each T3PKS, bacteriocin, and other (unknown) secondary metabolite gene clusters were identified in each *B. pumilus* strain. Only one NRPS cluster was identified each in strain SAFR-032, C4, and TUAT1, while two linear azol(in)e-containing peptides (LAPs) were identified exclusively in strain NCTC 10,337. The divergence between phylogenetic proximity of *B. pumilus* strains and distribution of BGCs found within their genomes is a strong indication of HGT which enables biosynthetic routes of diverse products in various biological niches. To investigate the distribution of niche-specific BGCs, we focus on gene clusters that are not shared by the strains of the same clade. The strain SF-4 carries two and three additional NRPS gene clusters compared to its most closely related strains SAFR-032 and 150a, respectively. Interestingly other strains that shared repeated patterns of BGCs either cluster within the same clade or share a specific habitat. The BGCs shared by only a few strains or strain-specific were probably gained via HGT and such clusters are potentially important for the competition and survival of a strain in a specific niche [51]. NRPs are “mega enzymes”, usually organized in many modules, each of which adds one amino acid to the growing peptide chain [52]. Besides the two common NRPS shared by all *B. pumilus* strains, two additional unique NRPS gene clusters were identified in strain SF-4 which showed 13% and 21% gene similarity with lipopeptide (BGC0000433) and lichenysin (BGC0000381) cluster, respectively [53,54]. Lichenysin is a more efficient cation chelator than surfactin while lipopeptides are known to inhibit the growth of phytopathogenic fungi and bacteria [53,55]. The gene cluster associated with terpene/siderophore (carotenoid) biosynthesis, represents a 50% similarity with known clusters (BGC0000645) from *Halobacillus halophilus* [56]. Terpenes/siderophores were previously reported to be involved in the biodegradation of heterocyclic compounds and also modulate cell membrane fluidity during oxidative stress [57].

**Figure 5:**
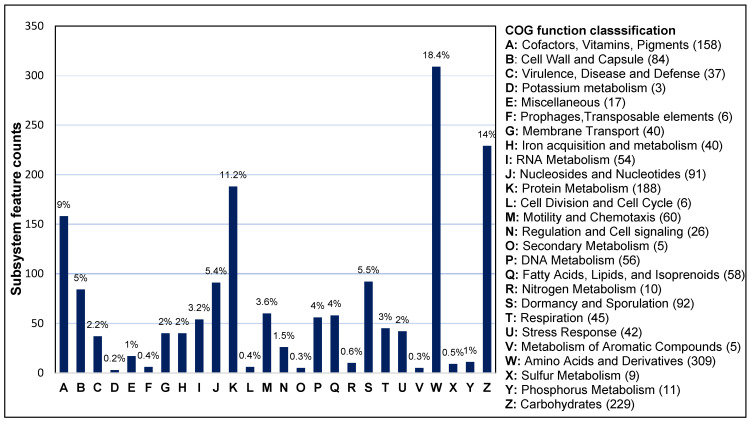
RAST subsystems categories and feature distribution of *B. pumilus* SF-4 genome. The percentage of category distribution is mentioned on each bar graph.

The genomic features of bacteria can be determined according to subsystem technology, which represents groups of genes performing specific biological functions in a structural complex. The strain SF-4 genome was analyzed via the RAST subsystem server and revealed 26 categories (Figure 5). Among these, the “amino acid and derivatives” category accounted for the largest number of 309 genes, followed by carbohydrate metabolism (*n* = 229); protein metabolism (*n* = 188); cofactor, vitamins and pigments (*n* = 158); dormancy and sporulation (*n* = 92); nucleosides and nucleotides (*n* = 91); and cell wall and capsule (*n* = 84). Regarding the 60 genes for motility and chemotaxis, 58 are related to fatty acid, lipids, and isoprenoids, 56 are linked with DNA metabolism and 54 were associated with RNA metabolism. Similarly, 42, 40, 11, and 10 genes were identified to be related to stress response, iron acquisition and metabolism, phosphorus metabolism, and nitrogen metabolism, respectively. The presence of numerous genes associated with these processes indicate that strain SF-4 might be resistant to stress condition and also harboring genes encoding plant growth-promoting metabolites, hence the strain could be developed and commercially formulated for field application to promote plant growth.

### 3.7. Comparative Genome Analysis

**Figure 6:**
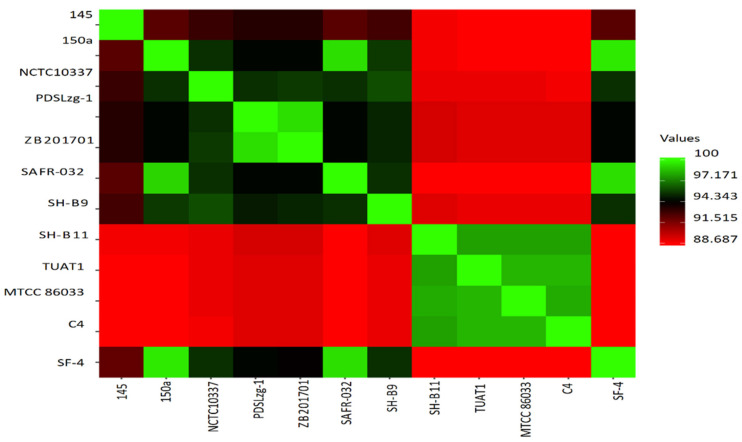
A heatmap representing the degree of similarity shared by 12 *B. pumilus* genomes based on average nucleotide identity (ANI) values. The map was derived from ANI matrix determined from low (dark red) to high (light green) similarities among genomes.

Genome comparison analyses based on average nucleotide identity revealed that *B. pumilus* strain SF-4 is most closely related to strains SAFR-032 and 150a, by sharing more than 99% identity (Figure 6). It is also notable that four strains, designated as SH-B11, TUAT1, MTCC B6033, and C4, cluster in a separate group (Figure 3) sharing more than 97% ANI with each other but less than 90% ANI with reference strains SAFR-032, 150a, as well as the newly isolated strain SF-4. ANI values indicate the relatedness or the genetic distance between two genomes and usually thresholds of more than 95% identity are used to delineate between separate species [58]. However, the applicable ANI thresholds may vary between different phylogenetic groups and are not yet precisely established for *B. pumilus* [59]. Therefore, we further performed digital DNA-DNA hybridization using the method [60]. GGDC analyses yielded DDH estimates greater than 79% between strain SF-4 and all 11 compared strains indicate that all 12 strains belong to the same species—i.e., *B. pumilus* (Supplementary File 2, Table S5).

**Figure 7:**
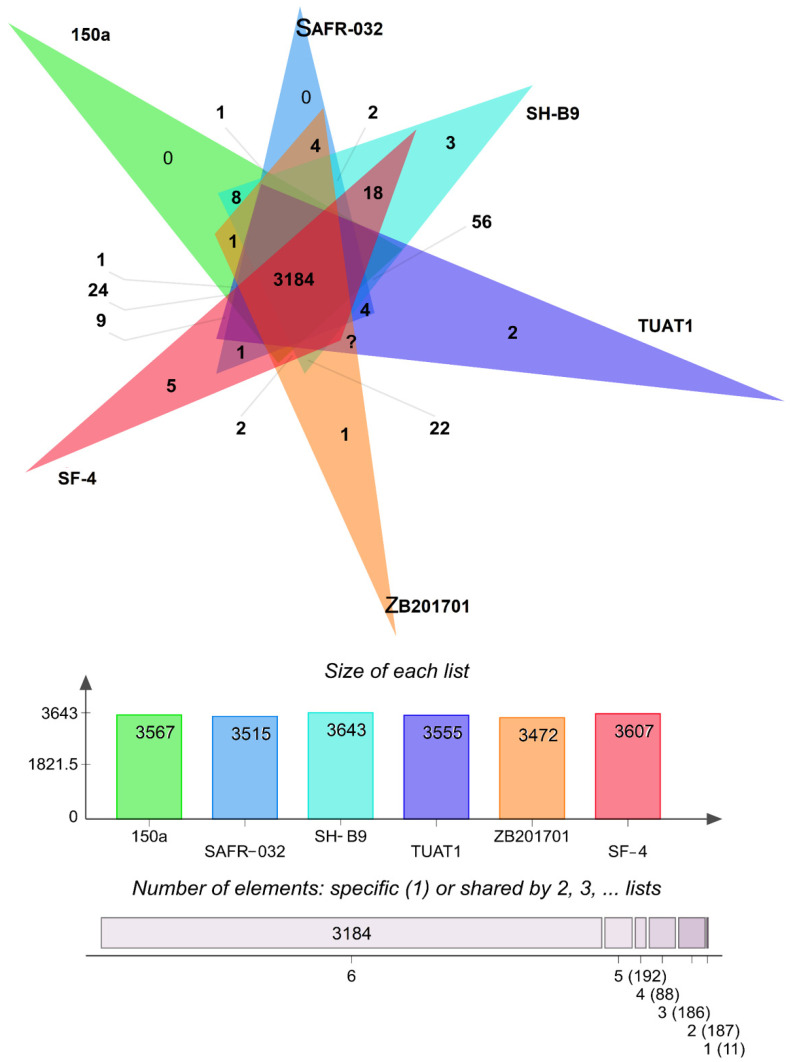
Proteome comparison among *B. pumilus* strains SF-4 (Soil field, Pakistan), TUAT1 (Japan), 150a (Sediment top, Mexico), SH-B11 (Sugar beet rhizosphere, Netherland), ZB201701 (China), and SAFR-032 (Spacecraft). The Venn diagram and bar chart represent the number of unique and shared orthologous genes for each strain.

Orthologous genes are genes that have evolved vertically from a single ancestral gene. Genome-wide comparison in different strains provide insight into gene structure, function, and evolution of genomes [61]. The COGs analysis of strain SF-4 was compared with other available *B. pumilus* genomes isolated from China (ZB201701), Mexico (150a), Japan (TUAT1), Netherland (SH-B11), and spacecraft (SAFR-032). The analysis revealed that strain SF-4 contains 3699 proteins, 3607 COGs, and 79 singletons. Among the 3607 COGs in strain SF-4, 3184 are shared by all strains and five COGs are specific to strain SF-4 (Figure 7). Functional enrichment analysis showed that the unique COGs in strain SF-4 are either associated with antibiotics biosynthesis processes (GO: 0017000) or with unknown functions. The exact biological role of these genes in strain SF-4 is unknown. Therefore, further investigation is required to understand the characteristics of these unique genes. The targeted 12 *B. pumilus* strains form a total of 3848 clusters, 669 orthologous clusters, and 3189 singletons.

### 3.8. Plant Growth-Promoting Traits

#### 3.8.1. Phosphate Solubilization

Phosphate is an essential component required for plant growth and development. However, the majority of phosphate in the soil is immobilized and not available to plants [62]. Some bacteria are capable of solubilizing the insoluble phosphate and making it available to plants. This solubilization of immobilized phosphate by bacteria is achieved via gluconic acid production and is facilitated by glucose dehydrogenase (*gdh*) [63,64]. We screened for and detected *gdh* genes in the strain SF-4 genome and also found nine additional genes associated with phosphorus metabolism and transportation (Supplementary File 2 Table S6). The presence of these genes indicates that *B. pumilus* SF-4 is capable of solubilizing inorganic phosphate into a soluble form and may be used as inoculants to enhance phosphate uptake by plants. Another rich source of soil phosphate is immobilized in the form of phosphonate that must be hydrolyzed before biological incorporation. Bacterial degradation of phosphonate to phosphate is carried out by products of the *phn* gene cluster, which consists of 14 genes named *phnC* to *phnP* [65]. The strain SF-4 contains three genes of phosphonate biodegradation pathway encoding for phosphodiesterase (*phnP*), alkyle phosphonate utilization protein, and acid phosphatase. The missing of few genes in the phosphonate gene cluster may be attributed to the occurrence of gene gain or loss events during the evolutionary process. The major transport system of phosphate is composed of phosphate-specific transporter (*pst*) previously reported in *B. subtilis* [66]. Here we also detected the *pst* operon composed of phosphate ABC transporter permeases (*pstABC*) in strain SF-4 genome.

#### 3.8.2. Iron Acquisition

Similar to phosphate, iron although present in the soil, however, is mainly unavailable for plant utilization. Siderophores are extracellular, low molecular weight chelators produced by bacteria under iron-deficient environments. Siderophores are mainly synthesized by NRPS and translocated via the outer membrane [67]. The biosynthetic gene cluster for siderophore (bacillobactin) in the genome of strain SF-4 is encoded by the *dhbABCEF* operon. Our comparative genome analysis revealed that seven more *B. pumilus* strains including 145, 150a, NCTC10337, PDSLzg-1, SH-B9, SHB11, and ZB101701 harbor this *dhb* operon. Previously, similar *dhb* clusters were identified in 4 plant-growth-promoting *B. cereus* strains [68]. Besides plant growth, iron is an essential micronutrient for bacterial growth and is involved in biofilm formation. Biofilm formation is an important factor contributing to colonization and may serve as a survival mechanism in stress conditions [69]. Therefore, along with BGCs we also focused on siderophore transporter genes in the SF-4 genome. Membrane spanning ABC transporters and membrane-bound substrate-binding proteins facilitate the uptake of siderophores in Gram-positive bacteria [70]. Correspondingly, Iron-hydroxamate ABC transporter clusters and ferric-hydroxamate gene clusters were identified in the SF-4 genome (Supplementary Table S6). Moreover, the stimulator of *dhbF*, tyrosine adenylation activity (*mbtH*) was also detected.

From a global perspective, biological control is considered a more eco-friendly and interesting complement to disease management. Nonetheless, various aspects of this strategy are not yet explored properly [71]. *B. pumilus* owns a major advantage over other biocontrol agents due to its spore-forming ability [7], which allows this bacterial strain to withstand a harsh ecological environment and simultaneously can fight against plant pathogen and produce plant growth-promoting metabolites.

## 4. Conclusions

In the present study, we isolated an antibacterial exhibiting *B. pumilus* strain SF-4 from a soil field and sequenced its genome, yielding a genome size of 3.77 Mb. Comparative genome analysis revealed selective expansion of niche-specific genome content in *B. pumilus*. The expended unique genes contribute to strain fitness and adaptability to various ecological niches. *B. pumilus* SF-4 harbors several beneficial gene clusters including antimicrobial metabolite and PGP gene that will provide cross-protection against phytopathogen and promote plant growth simultaneously. This study may paw a way to the application of *B. pumilus* strain as an alternative sustainable strategy to improve crop yield under unfavorable conditions. However, further transcriptomic and metabolomic analysis of strain SF-4 is required to confirm the association of the genes highlighted here with the indicated functional potential.

## Figures and Tables

**Table 1:** Characterization of prophages in *B.pumilus* strain SF-4 genome.

Region	Length	Completeness	Score	Proteins	Location	Prophage	GC %	Phage Components
1	59.9 kb	Complete	140	76	513064-572967	Brevib_Jimmer1 (NC_029104)	41.22	Capsid, integrase, terminase, tail, and head
2	19.5 kb	Incomplete	10	11	1680797-1700309	Bacill_SP_10 (NC_019487)	40.63	NA
3	27.3 kb	Incomplete	50	36	3559626-3586998	Brevib_Jimmer1 (NC_029104)	40.79	Tail, head, terminase
4	14 kb	Incomplete	30	16	2744188-2758208	Bacilli- SPbeta (NC_001884)	36.52	Tail and transposase
5	27.8 kb	Incomplete	50	30	3746776-3774609	Staphy_SPbeta_like (NC_029119)	48.27	Transposase and integrase

**Table 2:** Comparative genome features of 12 *B. pumilus* genomes.

Strain	Accession	Size (Mb)	GC%	Protein	rRNA	tRNA	Other RNA	Gene	Pseu	Contamination (%)	Completeness (%)	Source
SH-B9	CP011007.1	3.79	41.6	3727	24	81	5	3887	50	1.98	95.49	Sugar beet rhizosphere, The Netherlands
NCTC10337	LT906438.1	3.86	41.7	3775	24	81	5	3950	65	3.35	95.83	NCTC United Kingdom
145	CP027116.1	3.94	41.2	3880	24	81	5	4064	74	2.71	92.62	Sediment top, Mexico
SH-B11	CP010997.1	3.86	41.3	3776	24	81	5	3913	27	5.27	95.30	Sugar beet rhizosphere, Netherlands
MTCC B6033	CP007436.1	3.76	41.4	3708	24	81	5	3881	63	3.12	95.06	Culture collection Canada
150a	CP027034.	3.75	41.4	3643	24	82	5	3821	67	1.93	95.64	Sediment top, Mexico
TUAT1	AP014928.1	3.72	41.4	3688	24	81	5	3817	19	1.24	96.49	Field soil, Japan
SAFR-032	CP000813.4	3.7	41.3	3588	21	72	5	3764	78	0.17	98.49	Spacecraft
PDSLzg-1	CP016784.1	3.7	42.0	3600	24	81	5	3761	51	2.62	93.07	Oil Sands, China
ZB201701	CP029464.1	3.64	41.9	3545	24	81	5	3723	68	1.78	93.24	Rhizosphere soil, China
SF-4	CP047089.1	3.77	41.2	3669	10	75	5	3845	86	2.28	96.36	Soil field, Pakistan
C4	CP011109.1	3.66	41.4	3622	10	59	5	3728	32	1.42	94.74	Compost, Egypt

## Data Availability

The *B. pumilus* SF-4 genome sequence was deposited to NCBI and available under GenBank accession number CP047089.1. The Biosample and Bioproject were registered under accession number SAMN13526181 and PRJNA594265, respectively.

## Supplementary Materials

The following are available online at https://www.mdpi.com/article/10.3390/genes12071060/s1, Figure S1: Antibacterial activities of *B. pumilus* strain SF-4 against indicator ATCC strains, Table S1: Detail of genomic islands identified in *B. pumilus* SF-4 genome, Table S2: Pan-core genome analysis of *B. pumilus* strains, Table S3: Description of unique genome pool in *B. pumilus* strain SF-4, Table S4: Characterization of BGCs identified in *B. pumilus* SF-4 genome, Table S5: Genome-to-genome distance calculation (GGDC) values of *B. pumilus* strain SF-4 with other strains, Table S6: Plant growth promoting capabilities of *B. pumilus* strain SF-4.
