# Supplementary material for: Genome Mining and Comparative Genome Analysis Revealed Niche-Specific Genome Expansion in Antibacterial Bacillus pumilus Strain SF-4
Source: Genes (Basel). 2021 Jul 12;12(7):1060. doi: 10.3390/genes12071060 (PMC8303946; doi:10.3390/genes12071060)
Supplement: Supplementary file 1 [file genes-12-01060-s001.zip › Supply file 1 Genes rev.pdf]

# Genome mining and comparative genome analysis revealed niche-specific genome expansion in antibacterial *Bacillus pumilus* strain SF-4

**Sajid Iqbal [a], John Vollmers [b], Hussnain Ahmed Janjua [a*]**

[a] Department of Industrial Biotechnology, Atta-ur-Rahman School of Applied Biosciences (ASAB), National University of Sciences and Technology (NUST), H-12 Islamabad, Pakistan.
[b] Institute for Biological Interfaces 5 (IBG-5), Institute of Technology (KIT), 76344 Eggenstein-Leopoldshafen, Germany.
* Correspondence: Hussnain Ahmed Janjua; hussnain.janjua@asab.nust.edu.pk

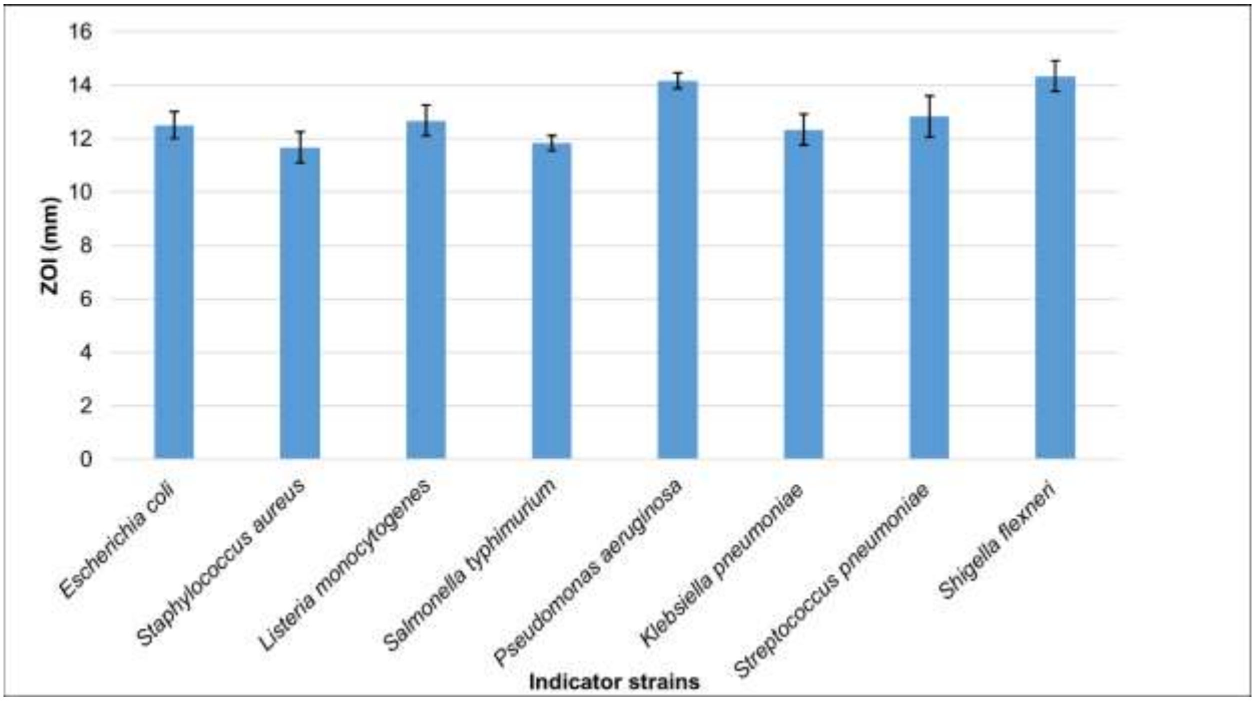


**Figure S1.** Antibacterial activities of *Bacillus pumilus* strain SF-4 against indicator ATCC strains. Errors bars indicate the standard deviation of triplicate experiments.
